# Applications of Antioxidant Nanoparticles in Immune-Mediated Inflammatory Diseases

**DOI:** 10.3390/antiox14091128

**Published:** 2025-09-18

**Authors:** Hong-Wei Shi, Bo-Cheng Yang, Yun-Qing Ren, Yi Xue

**Affiliations:** 1 Department of Radiology, The Children’s Hospital, Zhejiang University School of Medicine, National Clinical Research Center for Child Health, Hangzhou 310058, China; 18846794048@163.com; 2 Department of Plastic Surgery, Zhongshan Hospital Affiliated to Xiamen University, School of Medicine, Xiamen University, Xiamen 361005, China; 3 Department of Dermatology, The Children’s Hospital, Zhejiang University School of Medicine, National Clinical Research Center for Child Health, Hangzhou 310058, China

**Keywords:** antioxidant, immune, inflammatory, alopecia areata, multiple sclerosis

## Abstract

Immune-mediated inflammatory diseases (IMIDs) encompass a wide range of disorders, including autoimmune, acute, and chronic inflammatory conditions, which are often characterized by immune dysregulation and excessive oxidative stress. Oxidative stress plays a pivotal role in the initiation and progression of these diseases by promoting tissue damage and sustaining inflammation. However, conventional antioxidant therapies are limited by poor bioavailability, inadequate targeting, and short-lived efficacy. In recent years, nano-antioxidants have emerged as a promising therapeutic approach due to their enhanced stability, targeted delivery capabilities, and multifunctional therapeutic effects. This review provides a comprehensive overview of recent advances in the application of nano-antioxidants in the treatment of IMIDs. Their therapeutic roles are categorized into three major groups: autoimmune diseases, acute inflammatory diseases, and chronic inflammatory diseases. In autoimmune disorders such as alopecia areata and multiple sclerosis, nano-antioxidants have demonstrated the ability to reduce oxidative damage, modulate immune responses, and alleviate clinical symptoms. In acute inflammatory conditions, including acute kidney injury and acute liver injury, these nanomaterials exert protective effects by scavenging ROS, mitigating tissue injury, and restoring organ function. In chronic inflammatory diseases such as inflammatory bowel disease and ulcerative colitis, nano-antioxidants contribute to maintaining mucosal integrity, suppressing chronic inflammation, and improving therapeutic outcomes through localized delivery and sustained release. In summary, nano-antioxidants represent a novel and promising therapeutic strategy for the management of IMIDs. Their unique physicochemical properties offer significant advantages over traditional treatments. Further research is needed to optimize their delivery platforms, evaluate long-term safety, and facilitate clinical translation.

## 1. Introduction

Alopecia areata (AA), multiple sclerosis (MS), and other conditions—including inflammatory bowel disease (IBD), acute liver injury (ALI), acute kidney injury (AKI), and vitiligo—result from immune system dysregulation. Collectively termed immune-mediated inflammatory diseases (IMIDs), these disorders comprise autoimmune pathologies and span both chronic and acute inflammatory processes [1]. Among the diverse pathogenic mechanisms involved, oxidative stress has emerged as a pivotal contributor to both disease onset and progression [2,3]. Oxidative stress, resulting from an imbalance between reactive oxygen/nitrogen species and intrinsic antioxidant systems, contributes to tissue dysfunction and cellular injury [4]. Accumulating evidence links oxidative stress to immune activation and tissue damage, while also implicating it in the amplification of inflammatory responses, thereby establishing a self-perpetuating pathogenic cycle.

Antioxidants, due to their capacity to counteract oxidative stress, have garnered considerable interest as therapeutic candidates for IMIDs. Conventional small-molecule antioxidants—such as vitamin C, vitamin E, and glutathione—exhibit limited clinical efficacy, primarily owing to low bioavailability, poor tissue specificity, and rapid systemic clearance [5,6]. Recent advances in nanotechnology have enabled new approaches for enhancing antioxidant therapy. Nano-antioxidants, constructed from nanomaterials with high surface-to-volume ratios, favorable biocompatibility, and modifiable surface chemistry, demonstrate improved physicochemical stability, enhanced biological targeting, and elevated therapeutic efficiency [7]. Notably, several nanomaterials possess intrinsic enzyme-mimetic properties. For instance, iron oxide- and manganese-based nanoparticles emulate the catalytic activities of endogenous antioxidant enzymes, including superoxide dismutase (SOD) and catalase (CAT), thereby facilitating efficient elimination of ROS and RNS [8,9]. Curcumin, a natural polyphenol derived from *Curcuma longa*, exhibits potent antioxidant and anti-inflammatory activities. However, its clinical application remains constrained by poor aqueous solubility, rapid metabolic degradation, and limited oral bioavailability. To overcome these barriers, various nanocarrier-based delivery platforms have been developed—such as liposomes, polymeric nanoparticles, solid lipid nanoparticles, and metal–organic frameworks [10]. These formulations significantly enhance curcumin’s systemic stability and targeting efficiency, while simultaneously potentiating its antioxidative and immunomodulatory effects. Preclinical studies indicate that curcumin nanoformulations attenuate inflammation, mitigate tissue damage, and improve disease outcomes in autoimmune models such as MS, primarily through ROS scavenging [11]. Beyond curcumin, alternative nano-antioxidant systems exhibit therapeutic potential across multiple IMIDs. Gold nanoparticles (GNPs), owing to their superior biocompatibility and tunable physicochemical properties, have been widely investigated in MS and other immune-related conditions [12]. Experimental findings suggest that GNPs reduce oxidative damage and modulate inflammatory signaling, thereby limiting immune-mediated tissue injury [13,14]. In the context of acute inflammatory pathologies, nano-antioxidants also demonstrate promising efficacy. A recent study reported an ROS-responsive nanoplatform integrating mitochondrial-targeted cerium oxide nanoparticles with atorvastatin. This system enabled site-specific drug release in a septic AKI model, suppressed oxidative stress and inflammation, reduced tubular epithelial apoptosis, and preserved renal architecture [15]. In diseases like IBD, characterized by persistent inflammation, oxidative stress is deeply implicated in driving the pathological cascade leading to tissue damage. Nano-antioxidants have been shown to alleviate intestinal inflammation and reinforce epithelial barrier function by modulating ROS levels and regulating signaling cascades, including NF-κB and Nrf2. Furthermore, co-administration with probiotics enhances therapeutic effects through modulation of gut microbiota homeostasis [16]. Despite substantial progress, several obstacles impede the clinical translation of nano-antioxidants, including uncertainties regarding long-term biocompatibility, metabolic fate, and immunogenicity. Future efforts should emphasize the rational design of nanotherapeutic systems featuring controlled release, disease-specific targeting, and optimal safety profiles to accelerate their deployment in the management of IMIDs.

We summarize the current applications and mechanisms of nano-antioxidants in IMIDs in Table 1, Table 2 and Table 3. This review provides a comprehensive overview of recent advances in the application of nano-antioxidants in immune-mediated inflammatory diseases, with a particular focus on their roles in autoimmune, acute inflammatory, and chronic inflammatory conditions. The underlying mechanisms, therapeutic efficacy, and safety profiles of nano-antioxidants are critically examined, and future directions for their development and clinical translation are discussed.

## 2. Methods

Relevant studies discussing nano-antioxidants in IMIDs were identified through an extensive literature survey utilizing five core academic databases, including PubMed, Embase, Scopus, Web of Science, and Google Scholar, focusing on publications from January 2020 to June 2025. The literature search included publications available up to June 2025. Keywords and search phrases were carefully selected, including terms such as “nano-antioxidants”, “autoimmune diseases”, “acute inflammation”, “chronic inflammatory diseases”, “alopecia areata”, “multiple sclerosis”, “ulcerative colitis”, “periodontitis”, and “inflammatory bowel disease”. The search strategy focused on identifying studies that explored the application, mechanism, or therapeutic efficacy of nano-antioxidants in immune-related inflammatory conditions. Studies meeting the following criteria were included: (1) investigations that evaluated nano-antioxidant therapies related to autoimmune, acute, or chronic inflammatory diseases; (2) research involving nanoformulations designed to improve antioxidant delivery or bioactivity; (3) preclinical or clinical studies with reproducible methodologies and consistent outcome measures; and (4) experimental studies—either in vitro or in vivo—examining the antioxidant capacity or immune-modulating effects of nanomaterials. Exclusion criteria were as follows: (1) studies with inconclusive evidence regarding the therapeutic benefit of nano-antioxidants; (2) publications lacking rigorous experimental protocols or clearly defined results; (3) non-randomized or observational studies without proper controls; (4) articles not published in English; and (5) narrative reviews, opinion pieces, or book chapters without primary research data.

## 3. Nano-Antioxidants for Autoimmune Diseases

### 3.1. Alopecia Areata

Characterized by patchy, non-scarring hair loss primarily on the scalp and other hairy areas, AA represents a T cell-mediated autoimmune inflammatory disorder [44]. Though the etiological framework of AA has not been fully delineated, the impairment of immune privilege within hair follicles continues to dominate current pathogenic models [45]. In addition, genetic predisposition, hypersensitivity, dysbiosis of skin and gut microbiota, and psychological stress are also recognized as important contributing factors to disease onset. Epidemiological data from the United States estimate the prevalence of AA to be approximately 0.1–0.2% [46]; however, this may be an underestimation due to underreporting and limited sample representativeness. AA significantly impairs patients’ quality of life and remains a therapeutic challenge in clinical practice. In recent years, oxidative stress—defined as an imbalance between pro-oxidant and antioxidant defenses—has been increasingly implicated in the disruption of immune privilege, contributing significantly to the pathogenesis of AA [47].

AA patients exhibit elevated systemic oxidative stress, as evidenced by increased serum levels of advanced glycation end-products (AGEs) and advanced oxidation protein products, alongside decreased antioxidant indices such as ferric reducing antioxidant power (FRAP), paraoxonase-1, and lecithin–cholesterol acyltransferase. Notably, FRAP levels correlated positively with the extent of hair loss and negatively with C-reactive protein, indicating a close association between oxidative stress, inflammation, and disease severity [48]. Similarly, in a cohort of 40 patients with alopecia areata (AA), Sachdeva et al. [49] reported significantly elevated serum malondialdehyde (MDA) levels, indicative of heightened oxidative stress. Concurrently, both total antioxidant status (TAS) and SOD activity were markedly reduced compared to healthy controls, with the degree of reduction showing a clear correlation with disease severity. Moreover, impaired autophagy has been implicated in AA, as evidenced by reduced expression of autophagy-related proteins ATG5 and LC3B and increased expression of p62 in hair matrix tissues of AA patients. Concurrently, oxidative stress biomarkers—including MDA, AGEs, and ischemia-modified albumin (IMA)—are significantly elevated in serum and lesional skin, suggesting potential diagnostic and monitoring value [50]. Taskin et al. [51] further demonstrated significantly increased serum levels of nitric oxide (NO·), peroxynitrite (ONOO^−^), and nitric oxide synthase (NOS) activity in AA patients, indicating that nitrosative stress also contributes to AA pathophysiology. As ONOO^−^ represents a convergence point between oxidative and nitrosative stress, it may exacerbate oxidative injury. These findings highlight NOS-related parameters as potential biomarkers. Accumulated evidence from observational studies over the past two decades (2000–2024) indicates that patients with AA consistently exhibit elevated oxidative stress markers (e.g., OSI, MDA) and reduced antioxidant enzyme activity, including SOD and glutathione peroxidase, suggesting a compromised antioxidant defense system. Collectively, these findings underscore the central role of oxidative stress in AA pathogenesis and provide a theoretical foundation for antioxidant-based therapeutic strategies [52].

**Figure 1:**
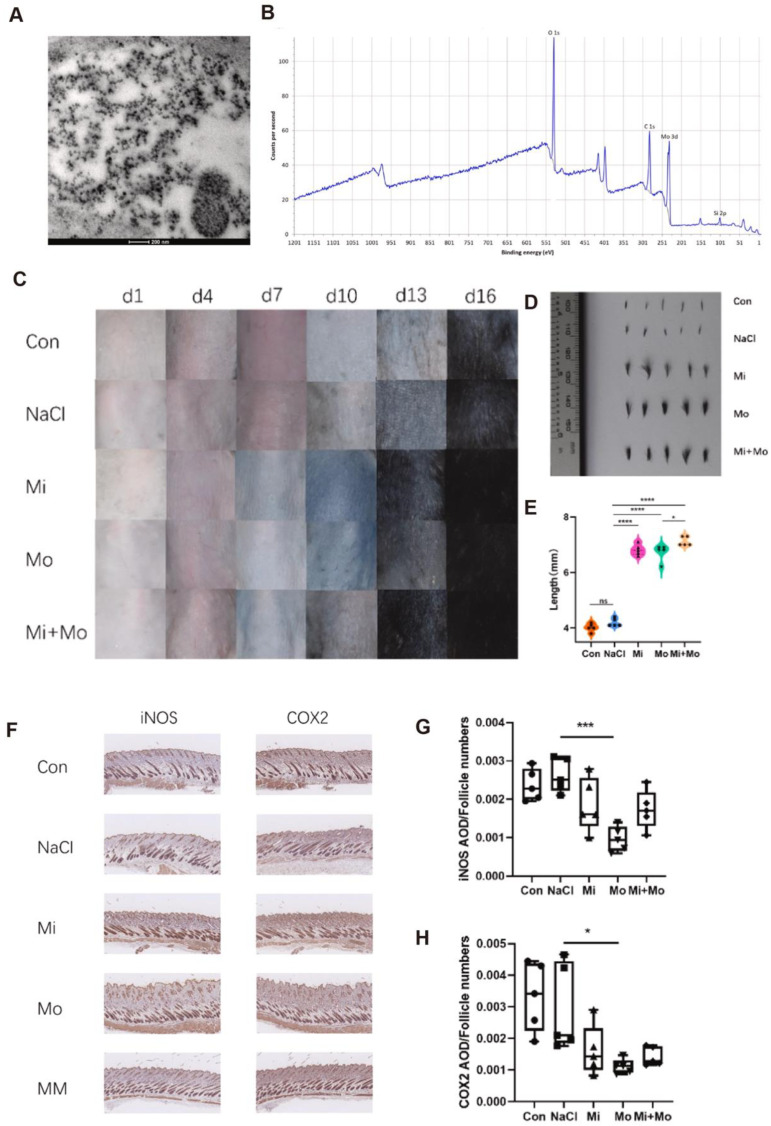
(**A**). TEM image showing Mo NPs distributed on mouse skin (scale bar: 200 nm). (**B**). XPS spectrum of Mo NPs, with O, C, and Si peaks as references. (**C**). Representative dorsal skin images on days 1, 4, 7, 10, 13, and 16 across treatment groups. (**D**). Hair length assessment on day 16 via representative photos. (**E**). Comparison of hair length among five groups on day 16. (**F**). Immunohistochemical staining of iNOS and COX2 in skin tissues (10×, scale bar: 100 μm) from Con, NaCl, Mi, Mo, and MM groups. (**G**,**H**). Quantitative AOD analysis of iNOS and COX2 per follicle. Mean ± SD values were obtained from five mice in each group (*n* = 5). * *p* < 0.05; *** *p* < 0.001 indicate statistical significance. Copyright 2024 Elsevier GmbH.

Meanwhile, localized drug delivery approaches—particularly microneedle patches—have attracted increasing attention. A hydrogel-based microneedle immunomodulatory system has been developed to deliver regulatory T cell (Treg)-associated factors, such as CCL22 and interleukin-2 (IL-2), to effectively recruit and expand Tregs within lesional areas, thereby re-establishing local immune tolerance. IL-2 functions as a crucial survival and proliferation factor for regulatory T cells (Tregs) and has been widely utilized to selectively expand Tregs in both experimental and clinical settings. In AA mouse models, this system significantly enhanced Treg infiltration, suppressed inflammatory signaling pathways, and promoted hair regrowth [53]. Additionally, soluble microneedle array patches (S-MAPs) composed of hyaluronic acid have been employed for the transdermal delivery of the Janus kinase (JAK) inhibitor baricitinib in a painless and noninvasive manner, thereby avoiding systemic adverse effects. In AA models, this strategy achieved significantly higher hair regrowth rates (82% vs. 31%) compared to oral administration [54]. Although baricitinib is not a direct antioxidant, it exerts indirect antioxidant effects by downregulating pro-inflammatory cytokines such as IL-6, IFN-γ, and TNF-α via JAK/STAT pathway inhibition, thereby reducing oxidative enzyme activity and ROS production, contributing to a combined anti-inflammatory and antioxidant effect [55]. In the realm of nanomaterials, Xiao et al. [17] examined the effects of molybdenum nanoparticles (Mo NPs) on AA progression through the modulation of oxidative stress. As a novel nano-antioxidant, Mo NPs possess a multivalent electronic structure and can promote hair regrowth by eliminating ROS, alleviating oxidative stress, and activating hair follicle stem cells. Characterization studies revealed that Mo NPs had an average diameter of approximately 50 nm (TEM, Figure 1A) and exhibited characteristic Mo 3d multivalence peaks (XPS, Figure 1B). In vivo, mice treated with Mo NPs exhibited visible hair regrowth as early as day 7 (Figure 1C), and hair shaft length was significantly greater than in controls (Figure 1D,E), with synergistic effects observed when combined with minoxidil. Mechanistically, Mo NPs significantly downregulated the expression of the oxidative stress markers iNOS and COX2 in hair follicle tissue (Figure 1F–H), further supporting their hair-promoting effect via antioxidative pathways. In conclusion, Mo NPs demonstrate excellent antioxidant activity and can act synergistically with conventional therapies such as minoxidil, offering a novel direction and translational potential for antioxidant-based treatment of AA. Future studies should explore their long-term efficacy and safety to facilitate clinical application.

### 3.2. Multiple Sclerosis

MS is characterized by immune-mediated damage targeting the central nervous system (CNS), leading to the formation of multifocal lesions in the brain and spinal cord, primarily involving demyelination and neuroinflammation. These lesions are predominantly located in the white matter and can be detected by magnetic resonance imaging. However, due to technical limitations, the identification of gray matter and cortical lesions remains challenging, posing a major difficulty in both diagnosis and treatment [56]. MS is frequently accompanied by psychiatric symptoms such as anxiety and depression, which significantly compromise patients’ quality of life and adherence to treatment. Reports indicate that between 2015 and 2022, the age- and sex-standardized incidence of MS showed a slight declining trend, ranging from 17.46 per 100,000 population in 2016 (95% CI: 17.12–17.80) to 15.65 per 100,000 in 2019 (95% CI: 15.34–15.97) [57]. The pathogenesis of MS is closely associated with immune-mediated inflammation and oxidative stress, the latter of which induces neuronal damage through ROS-related signaling pathways [58]. Fluctuations in the redox environment can trigger cell type-specific stress responses, disrupt metabolic homeostasis, and exacerbate disease progression. Therefore, therapeutic strategies targeting oxidative stress, particularly low-toxicity and multi-targeted nano-antioxidants, have emerged as a research hotspot in MS therapy.

To improve its chemical stability and enhance absorption, curcumin—a natural antioxidant—has been integrated into a range of nanocarrier technologies [59]. For example, dendritic nanocarriers (DNCs) based on curcumin have been shown to dose-dependently promote the differentiation of neural stem cells (NSCs) and oligodendrocyte precursor cells (OPCs) into oligodendrocytes, thereby enhancing remyelination capacity in vivo. DNCs also improve the survival and differentiation efficiency of transplanted NSCs, synergistically facilitating myelin repair [18]. In addition, a high-density lipoprotein-mimicking peptide-phospholipid scaffold (HPPS) has been utilized to deliver curcumin (Cur-HPPS) in a targeted manner. This system exerts anti-inflammatory and antioxidant effects by inhibiting the NF-κB pathway and reducing the expression of key mediators involved in immune cell recruitment, offering a novel direction for the precision treatment of MS [60]. Another study developed a system of iron oxide nanoparticles loaded with berberine (BBN-IONPs), which demonstrated superior neuroprotective and antioxidant effects in MS animal models compared to free berberine. This nanoplatform improved CPZ-induced cognitive impairment, elevated glutathione levels and total antioxidant capacity in brain tissues, reduced lipid peroxidation and inflammatory mediators, and modulated the expression of the key molecules MBP and MMP-9 and other key effector molecules [19].

**Figure 2:**
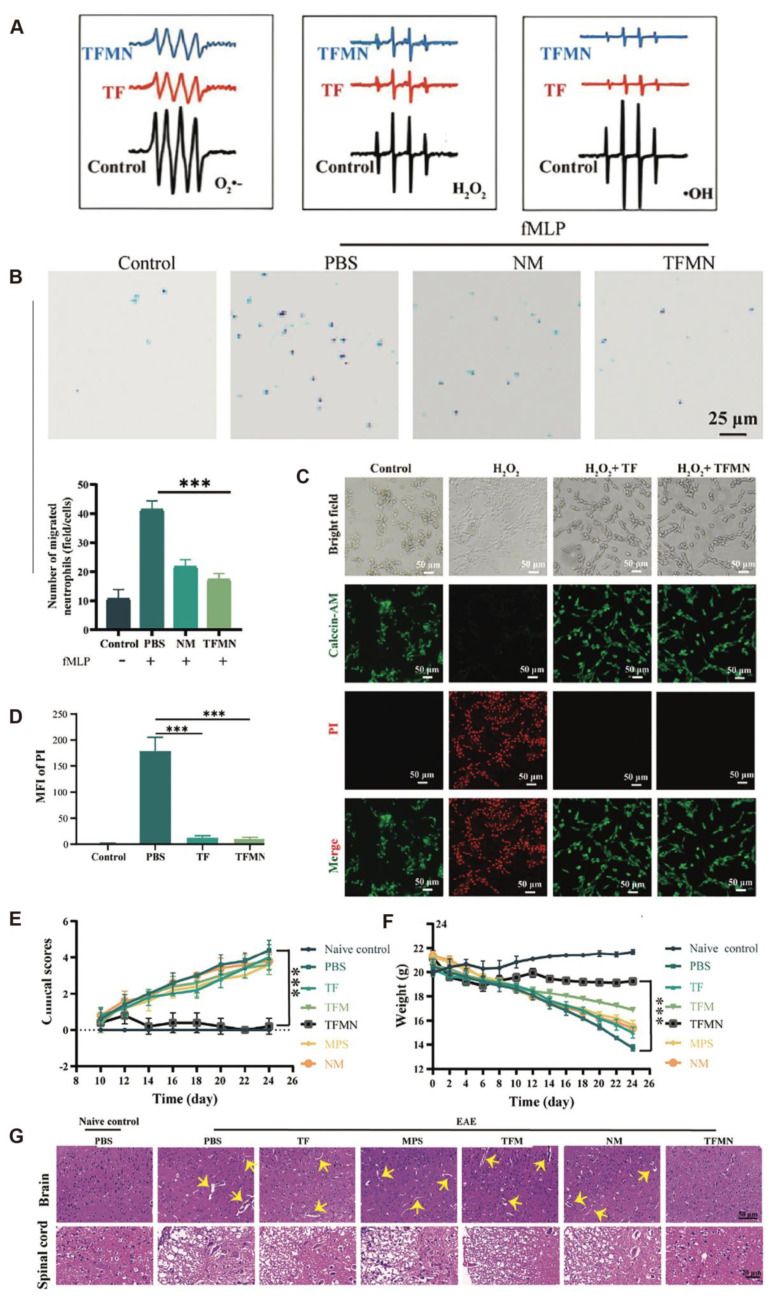
(**A**). Electron paramagnetic resonance (EPR) spectra of O_2_^•−^, H_2_O_2_, and ^•^OH in various treatment groups, with 5,5-dimethyl-1-pyrroline N-oxide (DMPO) employed as the spin-trapping reagent. (**B**). Representative images and quantification of neutrophil migration (*n* = 3). Scale bar: 25 μm. (**C**,**D**). Live/dead cell staining and corresponding quantification of propidium iodide (PI) fluorescence intensity in PC12 cells under different treatments. (**E**). Average clinical scores recorded for mice in each experimental group (*n* = 6). (**F**). Changes in body weight of EAE mice subjected to distinct treatment protocols (*n* = 6). (**G**). H&E-stained sections of brain and spinal cord tissues from EAE mice. Scale bars: 50 μm (brain) and 20 μm (spinal cord). Yellow arrows highlight areas of demyelination. Statistical significance assessed by one-way ANOVA followed by Tukey’s post hoc test, *** *p* < 0.001. Copyright 2024 Wiley-VCHGmbH.

In recent years, antioxidant strategies based on nanotechnology have shown broad application prospects in autoimmune diseases of the CNS. Low-toxicity magnesium hydride nanoparticles (MgH_2_) have also shown promising efficacy in MS mouse models, significantly alleviating anxiety- and depression-like behaviors and attenuating inflammatory demyelinating lesions [20]. The underlying mechanism may involve the promotion of M2-type microglial polarization, the inhibition of M1-type polarization, and the mitigation of oxidative stress and mitochondrial damage, suggesting their potential in treating MS-related neuropsychiatric disorders. Sanadgol et al. [61] showed that metformin facilitates the maturation of oligodendrocyte progenitor cells via the activation of the AMPK/mTOR signaling pathway while enhancing Nrf2-mediated antioxidant responses, thereby facilitating remyelination. Abdelalim et al. [21] developed a double-layered nanoemulsion system using lactoferrin and chitosan to encapsulate clobetasol propionate (CP), improving brain-targeted delivery and reducing systemic toxicity. The prepared nanoparticles exhibited stable physicochemical properties (mean particle size: ~220 nm; zeta potential: +30.23 mV) and an antioxidant capacity equivalent to 9.8 μM of vitamin C. Rezaeimanesh et al. [22] were the first to evaluate the effects of crocin–selenium nanoparticles (Cor@SeNs) on cognitive function and oxidative stress status in patients with MS. In a triple-blind, randomized controlled trial, 60 MS patients treated with Cor@SeNs for 12 weeks showed a marked enhancement in total antioxidant capacity (TAC; *p* = 0.01) and marked improvements in several cognitive assessment scores, including CVLT-II, CVLT-II-delay, and SDMT (all *p* < 0.01), indicating its potential in ameliorating MS-associated cognitive impairment. Yang et al. [23] developed a biomimetic nanosystem (TFMN) camouflaged with neutrophil membranes, which demonstrated multiple therapeutic effects in MS models. TFMN demonstrated potent scavenging activity against key reactive oxygen species, including O_2_^^•^−^, H_2_O_2_, and ^•^OH (Figure 2A), thereby reducing ROS-mediated neuronal injury, and significantly inhibited the migration of inflammatory cells across the blood–brain barrier (Figure 2B). In a PC12 cell oxidative injury model, TFMN significantly enhanced cell viability (Figure 2C–E), and in EAE mouse models, it lowered clinical scores, maintained stable body weight (Figure 2E,F), and mitigated inflammatory cell infiltration and demyelination in the brain and spinal cord (Figure 2G), indicating excellent safety and therapeutic potential. In summary, antioxidant strategies—particularly those based on multi-targeted nanomaterials—exhibit substantial advantages in the treatment of MS. These approaches not only alleviate inflammation and oxidative damage but also show potential in improving cognitive and psychiatric comorbidities, providing new perspectives and experimental foundations for the future development of individualized and precision therapies for MS.

## 4. Nano-Antioxidants for Acute Inflammatory Diseases

### 4.1. Acute Kidney Injury

The kidneys play a critical role in maintaining systemic homeostasis due to their abundant blood perfusion and high oxygen consumption, enabling efficient clearance of metabolic waste. However, these same characteristics also make the kidneys more susceptible to exposure to toxic substances and hypoxic conditions [62]. In AKI, renal function declines rapidly within a limited period, often leading to systemic complications that carry high morbidity and mortality. In the 2017 study population, the age-standardized incidence rates of AKI varied across regions, ranging from 12.9 to 14.9 cases per 1000 person-years [63]. Currently, no specific clinical therapies are available for AKI, highlighting the urgent need to develop effective therapeutic strategies. Accumulating evidence indicates that the excessive accumulation of reactive oxygen and nitrogen species (RONS) at injury sites is a key driver of AKI progression. However, due to the structural complexity, cellular heterogeneity, and specialized physiology of the kidneys, conventional antioxidant and anti-inflammatory small-molecule drugs exhibit limited efficacy, primarily due to poor targeting capability and adverse side effects [64].

**Figure 3:**
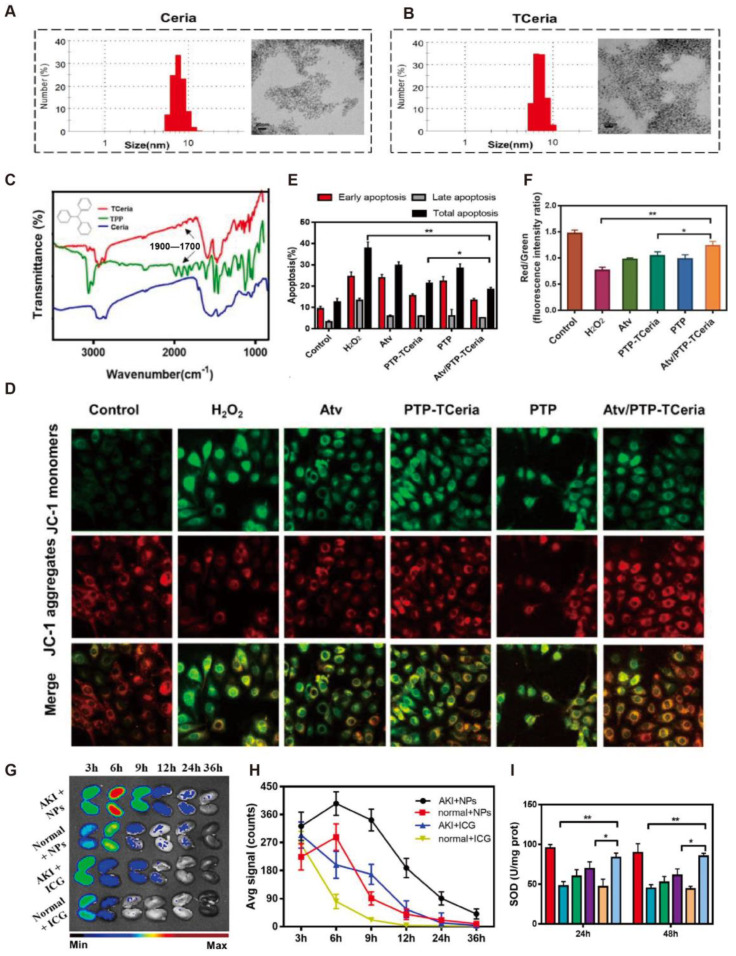
(**A**). Particle size distribution and transmission electron microscopy (TEM) images of Ceria NPs (scale bar: 20 nm). (**B**). Corresponding size distribution and TEM images of TCeria NPs (scale bar: 20 nm). (**C**). Fourier-transform infrared (FT-IR) spectra of TPP, Ceria NPs, and TCeria NPs. (**D**). Mitochondrial membrane potential (Δψm) assessed via confocal microscopy across treatment groups (scale bar: 20 μm). (**E**,**F**). Semi-quantitative analysis of Δψm alterations by flow cytometry and image-based quantification, respectively. Data are presented as mean ± SD (*n* = 3 per group). (**G**). Temporal changes in renal fluorescence intensity in AKI mice treated with ICG-loaded PTP-TCeria NPs versus free ICG. (**H**). Quantitative analysis of fluorescence intensity from panel. (**I**). Renal SOD levels measured at 24 and 48 h post-treatment. * *p* < 0.05, ** *p* < 0.01. CC BY 4.0.

To address these limitations, various nanomedicine-based antioxidant strategies have emerged. Gu et al. [24] synthesized a cerium–luteolin coordination nanocomposite (CeLutNCs), which effectively scavenges ROS, inhibits apoptosis, downregulates inflammatory cytokines, modulates macrophage activity, and suppresses NF-κB signaling. In both AKI models, CeLutNCs significantly improved organ function and reduced oxidative stress and inflammation, demonstrating strong therapeutic potential for ROS-related diseases. Zheng et al. [25] developed ultrasmall rhodium nanoparticles modified with L-serine (Rh-Ser, 2–4 nm), which mimic multiple antioxidant enzymes to efficiently eliminate RONS. Additionally, Rh-Ser generated oxygen at inflamed sites, alleviating hypoxia and suppressing inflammation, and increased the survival rate of AKI mice from 0% to 80%, outperforming traditional treatments. Yan et al. [26] designed a tetrahedral framework nucleic acid (tFNA)-based nanodrug delivery system (TTC), which specifically targets renal tubular cells and mitochondria, reduces oxidative stress, and suppresses apoptosis. In vitro and in vivo ischemia–reperfusion (I/R) models showed that TTC significantly ameliorated renal injury and restored renal function, indicating promising antioxidant-mediated therapeutic effects. Pan et al. [27] developed a pH-stable antioxidant nanoparticle system (EGA NPs), based on epigallocatechin gallate (EGCG) and 5-aminosalicylic acid (5-ASA). Through a “dissociation-reassembly” mechanism under acidic conditions, these particles self-assemble into stable nanoparticles with robust antioxidant and anti-inflammatory properties, suitable for both oral and intravenous administration. EGA NPs not only effectively scavenge ROS and mitigate oxidative stress but also serve as a drug delivery vehicle for anti-inflammatory agents such as curcumin, significantly enhancing their therapeutic efficacy in vivo. Jia et al. [28] proposed a supramolecular nanoplatform (Ser-HPEC) for AKI therapy, possessing ROS-responsive antioxidant capabilities. Leveraging a Kim-1-mediated targeting mechanism via L-serine, Ser-HPEC selectively accumulates at I/R-injured renal sites and, under high ROS conditions, releases two antioxidant molecules—ethyl caffeate and 4-hydroxybenzyl alcohol. This results in efficient ROS scavenging, attenuation of oxidative stress and acute inflammation, and preservation of renal structure and function. Furthermore, a cerium-based nanozyme system with mitochondrial targeting capacity was successfully constructed. Transmission electron microscopy (TEM) images revealed that unmodified Ceria nanoparticles (Ceria NPs) [15] had a uniform spherical morphology with an average diameter of 7.74 ± 1.35 nm, while triphenylphosphine (TPP)-modified TCeria NPs exhibited a slightly larger diameter of 8.16 ± 1.98 nm, retaining good shape and dispersity (Figure 3A,B). Fourier-transform infrared spectroscopy (FT-IR) confirmed successful TPP modification, with characteristic absorption peaks at 1700–1900 cm^−1^ corresponding to the aromatic structure of TPP (Figure 3C). Mitochondrial membrane potential (Δψm) was assessed using JC-1 dye. In H_2_O_2_-treated cells, a significant reduction in the red/green fluorescence ratio indicated mitochondrial depolarization, whereas treatment with Atv/PTP-TCeria NPs restored Δψm to near-normal levels (Figure 3D). Flow cytometry showed that this formulation significantly reduced H_2_O_2_-induced apoptosis (below 20%), outperforming both free drugs and empty carriers (Figure 3E). Quantitative fluorescence analysis further validated its mitochondrial protective effect (Figure 3F). In vivo imaging using near-infrared fluorescent dye (ICG)-labeled nanoparticles revealed sustained accumulation of Atv/PTP-TCeria NPs in the kidneys of AKI mice for over 36 h, indicating excellent passive targeting capability (Figure 3G). Quantitative analysis confirmed significantly higher fluorescence intensity in AKI kidneys compared to healthy controls, consistent with enhanced permeability and retention (EPR) effects (Figure 3H). Additionally, treatment with Atv/PTP-TCeria NPs significantly increased renal SOD activity, restoring redox homeostasis and demonstrating superior ROS-scavenging ability compared to free drugs and control groups (Figure 3I). In summary, the constructed Atv/PTP-TCeria NPs system possesses multiple advantages, including precise size control, effective mitochondrial targeting, ROS responsiveness, and synergistic drug release. These features enable it to significantly ameliorate AKI-associated pathological processes at both cellular and animal levels, offering a promising nanotherapeutic strategy for the treatment of AKI.

### 4.2. Acute Liver Injury

ALI is a clinically common and severe liver disorder that can be triggered by a wide range of etiologies, including drug overdose, viral infections, toxic exposures, pregnancy, autoimmune diseases, inherited metabolic disorders such as Wilson’s disease, or vascular abnormalities such as Budd–Chiari syndrome. ALI is characterized by extensive hepatocellular necrosis and rapid deterioration of liver function. In certain cases, it may rapidly progress to acute liver failure (ALF), a life-threatening condition with a mortality rate of up to 80% [65]. Oxidative stress, particularly the excessive accumulation of ROS in hepatic tissue, is considered a key pathogenic mechanism in the onset and progression of ALI. Although broad-spectrum antioxidants such as N-acetylcysteine (NAC) have demonstrated partial therapeutic benefits, their limited targeting capacity, low clearance efficiency, and poor bioavailability severely hinder clinical outcomes. Therefore, there is an urgent need to develop more efficient, liver-targeted ROS-scavenging strategies to provide novel therapeutic approaches for ALI management [66].

Given the central role of oxidative stress in ALI pathogenesis, the development of nanomedicine-based antioxidant platforms with high ROS-scavenging efficiency and hepatic specificity has emerged as a promising research direction. Several studies have focused on integrating natural antioxidant molecules with functional nanomaterials to enhance therapeutic efficacy. Luo et al. [29] fabricated phlorotannin-based nanoparticles (PT NPs) from seaweed-derived polyphenols via a glycine-assisted Mannich reaction to improve their stability and bioavailability. These nanoparticles exhibited robust ROS-scavenging activity in H_2_O_2_-induced HepG2 cell models and effectively alleviated oxidative. Similarly, Jang et al. [30] developed a catechin-based thermosensitive nanoformulation (CCN150), which significantly improved the solubility and stability of catechin while retaining its intracellular ROS-scavenging activity. CCN150 selectively accumulated at inflammation sites in vivo, attenuated systemic inflammation driven by ROS, and demonstrated excellent biocompatibility. In the context of acetaminophen (APAP)-induced liver injury, Song et al. [67] designed a carbon monoxide-releasing molecule, SMA/CORM2, which enhanced hepatic CO levels and suppressed oxidative stress. Mechanistically, SMA/CORM2 downregulated HIF-1α expression, promoted M2 polarization of macrophages, and activated the PI3K/Akt/mTOR pathway, thereby mitigating liver damage and facilitating tissue regeneration. Additionally, Mn_3_O_4_ nanozymes showed comparable efficacy to NAC at lower doses by simultaneously modulating the Nrf2 antioxidant axis and suppressing NF-κB-mediated inflammatory responses, offering synergistic antioxidative and anti-inflammatory effects [68].

**Figure 4:**
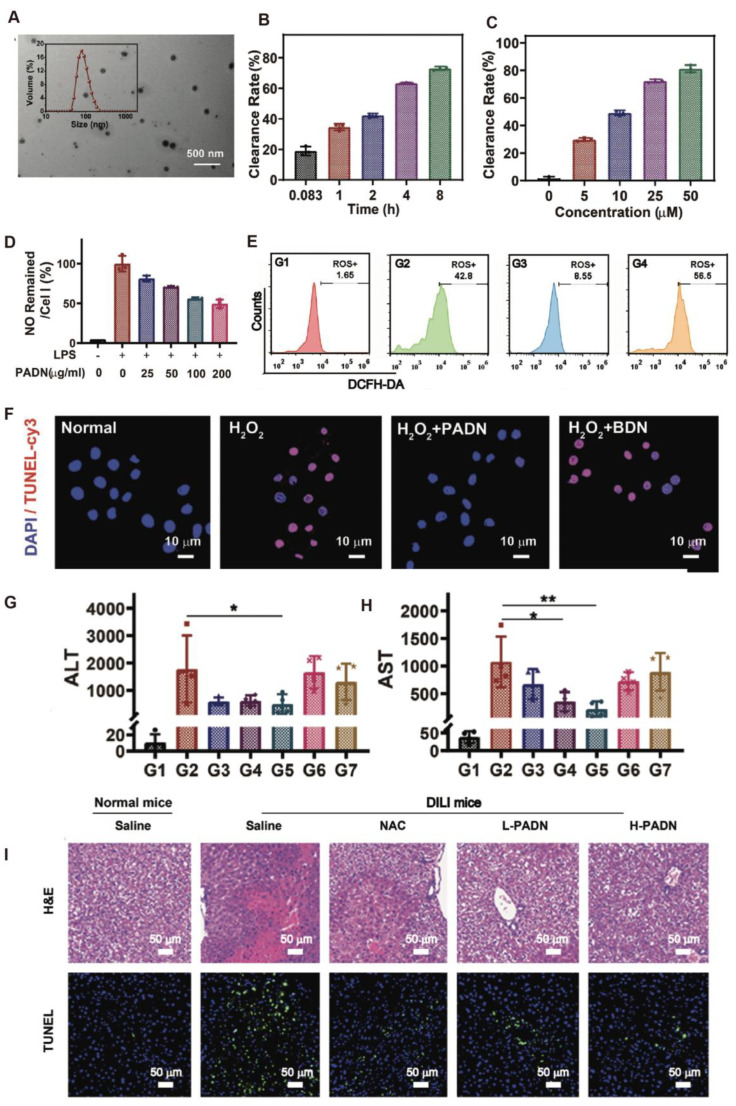
(**A**). TEM image and size distribution of PADN. (**B**). Time-dependent H_2_O_2_ scavenging activity of PADN. (**C**). Concentration-dependent H_2_O_2_ scavenging activity of PADN after 4 h incubation. (**D**). ^•^NO levels in RAW 264.7 cells (LPS-stimulated) treated with varying concentrations of PADN. (**E**). Analysis of intracellular ROS in cells (RAW 264.7) under different treatments by flow cytometry (G1: normal + saline; G2: DILI + saline; G3: DILI + NAC; G4: DILI + L-PADN; G5: DILI + H-PADN; G6: DILI + L-BDN; G7: DILI + H-BDN). (**F**). CLSM images of L02 cells stained with DAPI (blue) and TUNEL-Cy3 (red) under different treatments. (**G**,**H**). Serum ALT (**G**) and AST (**H**) levels in DILI mice receiving different pretreatments. (**I**). H&E and TUNEL staining of liver sections from each group. *t*-test, *n* = 4; * *p* < 0.05, ** *p* < 0.01. Copyright 2021 Wiley-VCH GmbH.

Curcumin, a natural compound widely used in traditional Chinese medicine, has shown hepatoprotective potential in APAP-induced ALI through mechanisms involving ROS scavenging, inhibition of lipid peroxidation, restoration of glutathione levels, and anti-apoptotic effects. The incorporation of curcumin into nanocarriers can significantly enhance its bioavailability and therapeutic performance. Wang et al. [31] further developed BSA-SNO nanoparticles capable of stably releasing nitric oxide (NO). These nanoparticles alleviated oxidative stress, improved hepatic microcirculation, and increased survival rates in ischemia–reperfusion and endotoxin-induced ALI models. Hepatoprotection was achieved via Nrf2/HO-1 pathway activation and suppression of NF-κB-driven inflammation and mitochondrial apoptosis. A saponin–gallic acid conjugate-based nano-delivery system also demonstrated promising anti-inflammatory effects in CCl_4_-induced ALI. By employing Zn/Ga@Gd-LDH as a pH-responsive carrier, the system enabled controlled drug release in the acidic microenvironment, suppressed p-p65 phosphorylation in the NF-κB pathway, and reduced CRP levels, exhibiting both in vitro and in vivo anti-inflammatory efficacy [69]. Yuan et al. [66] developed SeMC nanoparticles derived from the self-assembly of L-selenomethionine (SeMC), modified with glycyrrhizic acid for hepatic targeting. These nanoparticles significantly reduced lipid peroxidation, tissue vacuolation, and aminotransferase elevation in both APAP- and CCl_4_-induced ALI models while enhancing endogenous antioxidant enzyme activity, showing favorable biocompatibility and liver-protective potential. Moreover, Zhao et al. [32] proposed a responsive biomimetic antioxidant nanoplatform (PADN) composed of PEGylated L-DOPA precursors protected by boronic acid groups. Upon exposure to oxidative stress, PADN undergoes deprotection to release PEG–L-DOPA, which subsequently undergoes a melanogenesis-like transformation into a high-molecular-weight melanin-like polymer with broad-spectrum ROS-scavenging capability. The self-assembled PADN exhibited a particle size of approximately 106.6 nm (Figure 4A) and maintained excellent colloidal stability for over three months under physiological conditions. It efficiently eliminated multiple ROS types, including H_2_O_2_, in a time- and dose-dependent manner (Figure 4B,C). At the cellular level, PADN significantly suppressed NO production and intracellular ROS accumulation in LPS-activated RAW264.7 macrophages (Figure 4D,E) and reduced TUNEL-positive hepatocytes in an H_2_O_2_-induced apoptosis model (Figure 4F). In vivo, PADN (50 mg/kg) effectively decreased serum ALT and AST levels in APAP-induced ALI mice, outperforming NAC in liver function recovery (Figure 4G–H). Histological analyses revealed reduced necrosis, inflammatory infiltration, and central venous congestion in PADN-treated mice, with the liver architecture approaching normal (Figure 4I). Additionally, PADN possessed photoacoustic imaging capabilities, enabling real-time visualization of lesion sites. Collectively, PADN exemplifies an oxidative microenvironment-responsive nanoplatform capable of integrating efficient ROS scavenging, inflammation suppression, and hepatocyte protection. Its stable structure and biocompatibility make it a strong candidate for clinical translation of antioxidant nanotherapies in ALI treatment.

## 5. Nano-Antioxidants for Chronic Inflammatory Diseases

### 5.1. Periodontitis

The global health impact of periodontitis, a chronic inflammatory condition affecting millions, has shown a consistent upward trend over recent years. Data from 2011 to 2020 indicate that approximately 62% of dentate individuals worldwide are affected, with over half of the cases classified as moderate to severe and nearly one-quarter as severe [70]. Compared with epidemiological data from 1990 to 2010, the prevalence of severe periodontitis increased from 10.8% to 23.6% [71]. By 2019, the global number of individuals affected had reached 1.1 billion [72].

Beyond being a prevalent oral disease, periodontitis is closely associated with a range of systemic conditions. While microbial plaque serves as the primary etiological factor, the progression of the disease—especially in its severe or refractory forms—is predominantly driven by the host immune response. Neutrophil-mediated mechanisms, notably the overproduction of ROS, play a pivotal role in tissue degradation. Oxidative stress, induced by intrinsic factors (e.g., metabolic disorders) and external triggers (e.g., smoking), creates a pro-inflammatory microenvironment that exacerbates periodontal damage. Thus, antioxidant-based therapies, such as those incorporating resveratrol, are being explored as promising strategies for periodontal intervention [73]. Li et al. [74] developed a novel bioactive glass-based microneedle system loaded with antioxidant agents for the treatment of diabetes-associated periodontitis. This system comprises Zn/V co-doped bioactive glass nanoparticles, gallic acid, and oxidized methacrylated hyaluronic acid, offering both antioxidative and antibacterial properties. By penetrating the mucosal barrier, the microneedles effectively deliver active agents into gingival tissues, enhancing retention and bioavailability. In diabetic periodontitis rat models, the system significantly promoted alveolar bone regeneration and alleviated local inflammation via suppression of the JAK-STAT and NF-κB signaling pathways. Yang et al. [33] constructed a mesoporous bioactive glass-based nanocarrier system for quercetin delivery, addressing its poor water solubility and limited bioavailability. This sustained-release platform promoted both osteogenesis and angiogenesis in experimental periodontitis-related bone defects. Additionally, it modulated the local immune microenvironment by regulating macrophage polarization. Mechanistically, quercetin acted through the miR-21a-5p/PDCD4/NF-κB axis to restore bone immune homeostasis. Similarly, Wang et al. [75] incorporated quercetin into octahedral nanoceria structures to enhance its antioxidative and immunomodulatory effects. The resulting nanocomposite effectively scavenged ROS, inhibited pro-inflammatory M1 macrophage polarization, and promoted the reparative M2 phenotype, enabling a smooth transition from inflammation to regeneration. This shift was evidenced by an increased M2/M1 polarization ratio, along with a rebalanced inflammatory profile in vivo, underscoring its therapeutic potential for managing periodontal inflammation.

**Figure 5:**
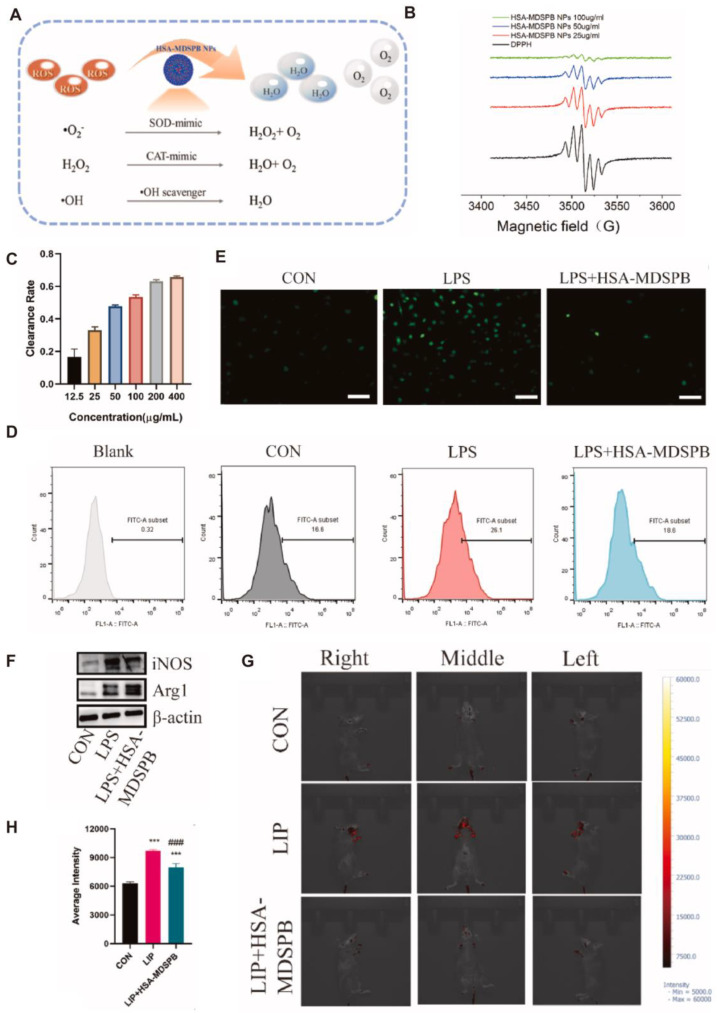
(**A**). Schematic diagram illustrating the antioxidative mechanism of HSA-MDSPB nanoparticles. (**B**,**C**). Assessment of the overall antioxidant activity of HSA-MDSPB NPs using DPPH and ABTS assays. (**D**). Fluorescence intensity analysis of MGF under different treatment conditions using DCFH-DA staining. (**E**). Representative fluorescence images of BMDMs stained with DCFH-DA (green) following various treatments (scale bar = 100 μm). (**F**). Western blot analysis showing protein expression in BMDMs under different treatment conditions. (**G**,**H**). In vivo imaging and quantitative evaluation of ROS levels in periodontal tissues on day 7 using the L012 probe. Statistical significance: *p* < 0.05, *p* < 0.001 (***, ###). CC BY 4.0.

To address oxidative stress-induced tissue injury in chronic periodontitis, Xin et al. [34] developed melatonin-derived carbon dots (MT-CDs) with excellent water solubility, biocompatibility, and ROS-scavenging capacity. MT-CDs stabilized mitochondrial function, mitigated oxidative cellular damage, and suppressed pro-inflammatory cytokine production. In a mouse model of periodontitis, MT-CDs reduced alveolar bone resorption and osteoclast activity. Cao et al. [35] designed a dual-polymer functionalized melanin-silver nanocomposite (P/D-MNP-Ag) with sequential therapeutic capabilities. One polymer becomes more cationic in acidic conditions to enhance membrane penetration, while the phosphate-rich polymer chelates Ca^2+^, promoting adhesion to hydroxyapatite surfaces. Initially, the system enables Ag^+^-mediated photothermal antibacterial activity; subsequently, as silver ions deplete, melanin’s intrinsic antioxidative properties are restored, allowing ROS clearance in inflamed regions. Animal studies confirmed its ability to disrupt biofilms, attenuate inflammation, and reduce alveolar bone loss. Moreover, Cao et al. [36] developed negatively charged human serum albumin-crosslinked, manganese-doped Prussian blue nanoparticles (HSA-MDSPB NPs) capable of broadly scavenging ROS species—including superoxide anions (O_2_^−^), hydroxyl radicals (^•^OH), hydrogen peroxide (H_2_O_2_), and singlet oxygen (^1^O_2_). As shown in Figure 5A–C, the nanoparticles exhibited strong radical-neutralizing capacity in DPPH and ABTS assays, achieving over 60% scavenging efficiency at 200 μg/mL. In vitro, they significantly reduced LPS-induced oxidative damage in periodontal cells: fluorescence analysis (Figure 5D,E) demonstrated decreased ROS levels in mouse gingival fibroblasts and bone marrow-derived macrophages (BMDMs). Additionally, HSA-MDSPB NPs facilitated macrophage repolarization, favoring an anti-inflammatory M2 profile over the pro-inflammatory M1 state (Figure 5F), thus promoting tissue repair. In vivo, using a ligature-induced periodontitis model (Figure 5G,H), these nanoparticles markedly reduced ROS accumulation, downregulated pro-inflammatory cytokines (e.g., IL-1β, TNF-α), and mitigated alveolar bone loss. In summary, by integrating ROS scavenging, immunomodulation, and osteogenesis promotion, HSA-MDSPB NPs—and related nanoplatforms—present a promising nanotherapeutic strategy for managing periodontitis, particularly under oxidative stress-driven pathological conditions.

### 5.2. Ulcerative Colitis

IBD, a chronic immune-driven condition targeting the gastrointestinal tract, primarily includes Crohn’s disease (CD) and ulcerative colitis (UC). In 2021, the global age-standardized incidence rate among adolescents and young adults reached a notable level (4.08 per 100,000 population) [76]. Although the precise etiology of IBD remains unclear, dysregulation of intestinal immune responses is widely acknowledged as a central mechanism. The dysregulated production of ROS and RNS under oxidative stress conditions significantly accelerates IBD development [3]. These reactive species can compromise intestinal epithelial integrity and activate pro-inflammatory signaling pathways, thereby promoting the progression of inflammation.

UC, one of the most prevalent forms of IBD, is characterized by a complex pathogenesis, multifactorial etiology, and recurrent clinical course. Current treatment strategies face significant limitations, including suboptimal efficacy, adverse effects, and high relapse rates. Thus, the development of novel therapeutic approaches with high efficiency, targeted delivery, and minimal toxicity has become a research priority.

Recent advances highlight the therapeutic promise of nano-antioxidants in UC, attributed to their targeted delivery, high loading efficiency, and diverse bioactivities. For instance, Ye et al. [77] developed selenium nanoparticles modified with Eucommia ulmoides polysaccharides (EUP-SeNPs), which significantly alleviated DSS-induced UC in mice by restoring colonic histology, enhancing mucosal barrier function, reducing pro-inflammatory cytokines, and modulating gut microbiota. Moreover, EUP-SeNPs exerted anti-inflammatory effects via suppression of the TLR4/NF-κB signaling pathway and improved antioxidant capacity in colon tissues. In further studies, EUP-SeNPs were dually functionalized with somatostatin (SST) and manno-oligosaccharides (MOSs) for epithelial–macrophage dual-targeting and encapsulated within alginate-based microspheres (SA/SST/MOS@EUP-SeNPs) to improve oral stability and intestinal localization [37]. This system enabled targeted accumulation at inflamed sites, enhanced antioxidative and anti-inflammatory efficacy, promoted mucosal barrier repair, and effectively relieved UC symptoms. To address the limitations of traditional enema therapy—such as short retention time and systemic toxicity—thiolated anionic nanoliposomes were constructed to deliver the natural antioxidant gallic acid. This system exhibited enhanced mucosal adhesion at inflamed colonic sites, thereby prolonging drug residence time and enhancing local therapeutic effects. In vivo experiments demonstrated that this nanocarrier significantly inhibited NF-κB, HIF-1α, and MMP-9 expression, reduced neutrophil infiltration, and promoted MUC2 expression and mucosal repair [38]. Another strategy utilized ROS-responsive nanocarriers based on diselenide-modified carboxymethyl cellulose (CMC) to deliver curcumin. This platform displayed excellent biocompatibility and ROS-triggered release. Through electrostatic interaction with cationic proteins in inflamed tissues, the system achieved targeted delivery and effectively scavenged ROS, restored mucosal integrity, and regulated gut microbiota composition to mitigate UC pathology [39].

**Figure 6:**
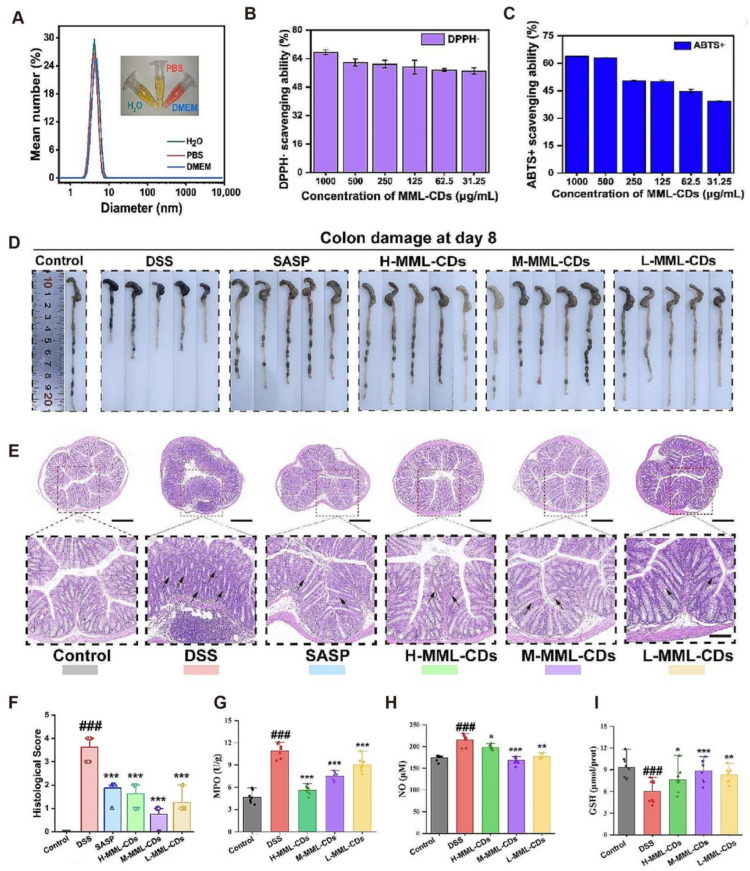
(**A**) DLS results of MML-CDs in different media (water, PBS, DMEM). (**B**,**C**) Free radical scavenging by MML-CDs (*n* = 6): (**B**) DPPH·; (**C**) ABTS^+^. (**D**) Colon morphology in each treatment group. (**E**) Colon length measurements (*n* = 5) (top: 40×, scale bar: 1 cm; bottom: 400×, scale bar: 100 μm). (**F**) H&E-stained colon sections. (**G**–**I**) Oxidative stress markers after oral MML-CDs in UC mice: (**G**) MPO, (**H**) NO, and (**I**) GSH (*n* = 8). Data were analyzed using the Kruskal–Wallis test or one-way ANOVA with Tukey’s correction. (### *p* < 0.001 vs. control; * *p* < 0.05, ** *p* < 0.01, *** *p* < 0.001 vs. DSS group). CC BY 4.0.

To overcome the low bioavailability of the resveratrol analog pterostilbene (PSB), researchers developed folate-modified, ROS-responsive nanoparticles (PSB@NP-FA) with an average size of ~231 nm. In inflammatory environments, PSB was released in a controlled manner, allowing efficient uptake by macrophages and epithelial cells. This formulation modulated dendritic cell function, promoted M2 polarization of macrophages, and regulated T cell infiltration. When co-administered with dexamethasone, it showed synergistic therapeutic benefits [40]. In the field of nanozymes, researchers constructed a polyethylene glycol-modified 2D Mo_3_Se_4_ nanosheet nanozyme (PMNFs), which mimicked multiple enzymatic activities, including peroxidase, glutathione peroxidase, superoxide dismutase, and catalase. In DSS-induced UC models, PMNFs efficiently scavenged ROS and improved colonic tissue damage. Mechanistically, they activated the Nrf2–Keap1 antioxidant pathway, inhibited TLR4/NF-κB-mediated inflammation, and protected tight junction proteins and MUC2 expression, thereby preserving intestinal barrier function [41]. Wei et al. [42] designed a pH-responsive, hyaluronic acid-modified nanoplatform (HA-L-Arg-CO_2_@NPs) for pterostilbene delivery. Upon encountering lysosomal acidic environments, the nanocarriers released CO_2_ to generate a “nanobomb” effect, promoting cytosolic drug delivery and site-specific accumulation. This system exhibited favorable colonic targeting and biocompatibility, significantly downregulated inflammatory mediators, restored barrier permeability, and effectively alleviated UC symptoms. Inspired by traditional Chinese medicine, Wu et al. [43] developed a novel functional carbon dot (MML-CD) synthesized via hydrothermal treatment of magnetite and Medicated Leaven. These ultrasmall nanoparticles (~3.2 nm) possess Fe-rich surfaces, excellent gastrointestinal stability, potent ROS-scavenging capacity, and high biocompatibility. In a UC mouse model, MML-CDs demonstrated promising therapeutic efficacy. They maintained physicochemical stability in various solutions, simulated digestive environments (Figure 6A), and displayed dose-dependent antioxidant activity against DPPH^•^ and ABTS+^•^ (Figure 6B,C). MML-CDs significantly improved symptoms such as bloody diarrhea, restored colon length, and alleviated tissue damage and inflammation (Figure 6D). Furthermore, they promoted hemostasis, reduced bleeding time and volume and prolonged APTT and PT, indicating regulation of coagulation responses (Figure 6E–I). Collectively, these findings suggest that MML-CDs offer a novel oral nano-antioxidant strategy for UC treatment through multi-modal mechanisms including antioxidation, anti-inflammation, and hemostatic enhancement. In summary, nano-antioxidant strategies employing targeted delivery, functional modification, and responsive release show significant promise in UC therapy. These systems enable precise accumulation at inflamed sites, reinforce antioxidative and anti-inflammatory defenses, and modulate mucosal immunity and barrier function—offering new opportunities for effective clinical translation.

### 5.3. Crohn’s Disease

CD is one of the major subtypes of IBD, and its global incidence has been steadily rising since 2000, with a prevalence of up to 1 in 200 individuals in Western countries. A prospective population-based cohort study conducted in Hungary between 2007 and 2018 reported an average annual incidence of CD at 9.9 per 100,000 population (95% CI: 9.0–10.9) [78]. CD can affect any part of the gastrointestinal tract and is characterized by segmental, chronic, relapsing, and transmural inflammation. Clinical manifestations include abdominal pain, diarrhea, bowel obstruction, and perianal lesions. While the exact cause of CD is not fully understood, substantial evidence points to the critical roles of genetic susceptibility, oxidative stress, immune dysregulation, and environmental stimuli in driving its onset and development [79]. Among these factors, oxidative stress plays a key role in IBD, especially in UC [80]. Multiple meta-analyses have confirmed the presence of significant oxidative damage in UC patients, particularly during active disease phases. This is characterized by elevated levels of lipid peroxidation products (e.g., MDA, 8-iso-prostaglandin F2α), and protein oxidation markers (e.g., advanced oxidation protein products) [81].

In this context, antioxidant-based therapeutic strategies have emerged as promising interventions for the prevention and treatment of CD. Crohn’s disease develops through multifactorial interactions among genetic, immunological, microbial, and environmental influences, wherein oxidative stress caused by disrupted redox balance plays a central pathogenic role. Both endogenous and exogenous antioxidants can neutralize ROS and attenuate oxidative damage, thereby modulating intestinal inflammation and serving as important adjuvant approaches to mitigate disease progression and improve clinical outcomes [82]. Chen et al. [83] systematically investigated the associations between genetically predicted levels of antioxidants, minerals, and vitamins and the risk of Crohn’s disease (CD) and ulcerative colitis (UC). Although not directly addressing immune metabolism, their findings revealed that specific micronutrient levels may influence disease susceptibility through oxidative stress and mitochondrial pathways. Furthermore, mechanistic studies highlighted the role of AFG3L2 in preserving mitochondrial integrity and enhancing antioxidant signaling via the PPARA/GPX4 axis, suggesting potential links between metabolic stress responses and intestinal inflammation [84].

Nanocarrier-based antioxidant therapies have emerged as a promising avenue for Crohn’s disease treatment in recent years [85]. Ibrahim et al. [86] developed a multi-strain probiotic formulation encapsulated in a nanoparticle delivery system (BBLNPs), comprising *Bifidobacterium breve*, *Bacillus coagulans*, and *Lactobacillus plantarum*. This formulation significantly improved inflammation in mouse model. BBLNPs effectively reduced oxidative stress markers (e.g., MDA, ROS, H_2_O_2_) and enhanced the activity of antioxidant enzymes (SOD, CAT, GSH-Px), thereby exhibiting synergistic regulation of oxidative stress, inflammation, autophagy, and metabolic pathways and showing substantial therapeutic potential. Mohanbhai et al. [87] proposed a colon-targeted drug delivery system encapsulating melatonin within chitosan nanoparticles, further coated with Eudragit-S-100 to achieve colonic release upon oral administration. In an LPS-induced macrophage model, this system demonstrated strong nitric oxide-scavenging ability. In a CD mouse model, it alleviated tissue inflammation, protected mucosal structure, reduced goblet cell loss, and decreased immune cell infiltration, indicating both antioxidant and anti-inflammatory efficacy. Zhu et al. [88] designed a selenium-enriched Prussian blue nanozyme (Se-HMPB) with multi-enzyme mimetic activities, capable of efficiently scavenging ROS at inflammatory sites and exhibiting pronounced antioxidant effects. The selenium component endowed the nanozyme with glutathione peroxidase-like activity, enabling inhibition of ferroptosis and reduction in lipid peroxidation in intestinal epithelial cells. Furthermore, Se-HMPB promoted intestinal barrier repair and regulated T cell differentiation, thereby strengthening immune barrier function and demonstrating favorable therapeutic efficacy in CD models. Shah et al. [89] synthesized a novel nitric oxide (NO)-releasing nano-antioxidant, S-nitrosoglutathione–alginate (SNA), constructed via conjugation and nitrosation of glutathione with alginate. This nanoformulation exhibited high NO loading capacity and sustained-release properties, offering a new antioxidant therapeutic option with potential for oral delivery in CD treatment.

**Figure 7:**
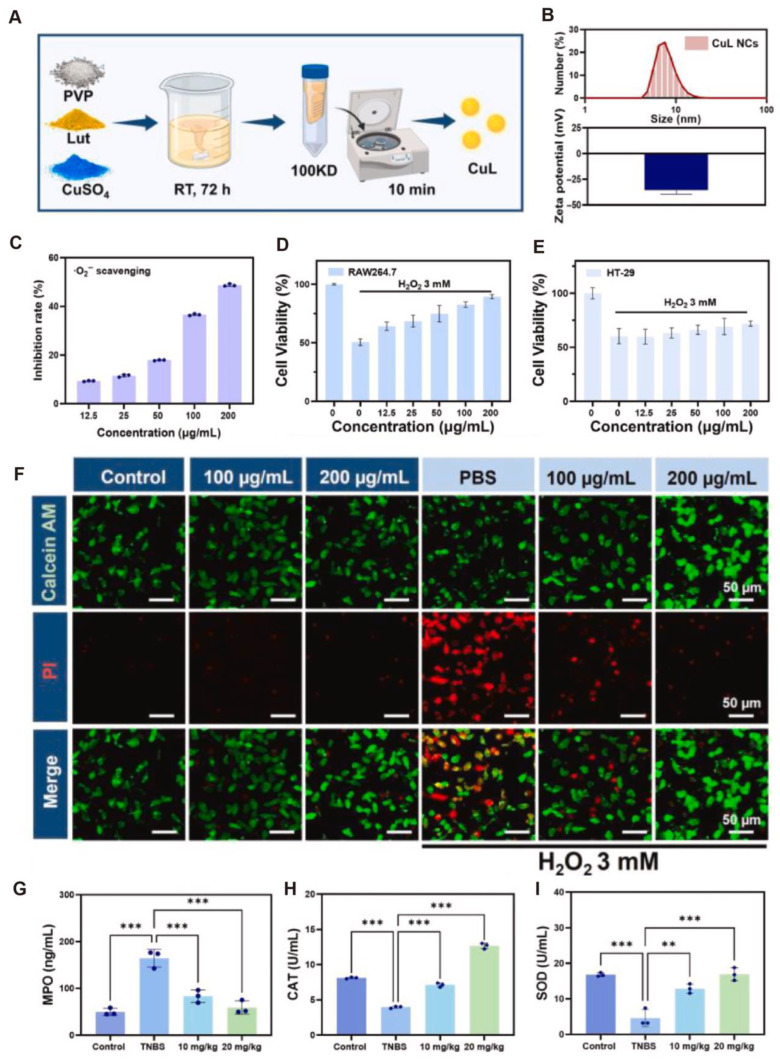
(**A**). Schematic illustration of the synthesis of CuL NCs. (**B**). Characterization of CuL NCs by dynamic light scattering (DLS) and zeta potential analysis. (**C**). Evaluation of concentration-dependent superoxide anion (O_2_^•−^)-scavenging activity. (**D**,**E**). Assessment of cell viability in RAW264.7 and HT-29 cells under H_2_O_2_-induced oxidative stress after CuL NCs treatment. (**F**). Fluorescence imaging of cell viability following H_2_O_2_ exposure with or without CuL NC pretreatment. (**G**). Measurement of MDA levels in colonic tissues. (**H**). Detection of myeloperoxidase (MPO) activity in colonic tissues. (**I**). Analysis of CAT activity in colonic tissues. ** *p* < 0.01, *** *p* < 0.001. CC BY 4.0.

Additionally, Fu et al. [90] developed a copper–flavonoid nanocomposite (CuL NCs) with multifunctional properties. The composite was synthesized through metal–polyphenol coordination self-assembly between luteolin and Cu^2+^ ions, forming uniformly sized nanoparticles (~7.58 ± 0.90 nm) with excellent dispersibility and colloidal stability in aqueous solution (Figure 7A,B). CuL NCs also exhibited strong free radical-scavenging capacity, particularly against superoxide anions (O_2_^•−^), in a dose-dependent manner, outperforming individual components and their physical mixture (Figure 7C). In H_2_O_2_-induced oxidative stress cell models, CuL NCs significantly improved the viability of cells (RAW264.7; HT-29), fully restoring cell viability at 200 μg/mL and demonstrating robust cytoprotective effects (Figure 7D–F). In a TNBS-induced CD mouse model, CuL NCs markedly reduced MDA levels and MPO activity in colonic tissues while restoring CAT enzyme activity (Figure 7G–I), indicating effective mitigation of CD-associated oxidative injury. In summary, CuL NCs possess both SOD-like and CAT-like activities, efficiently scavenging ROS and ameliorating the oxidative microenvironment. Through a tri-modal mechanism encompassing antioxidant, anti-inflammatory, and barrier-protective actions, they exert multidimensional therapeutic effects in CD. These findings provide a strong theoretical foundation and emerging evidence for the application of nano-antioxidants in CD and other chronic inflammatory bowel diseases.

## 6. Others

In addition to their well-documented applications in autoimmune, acute, and chronic inflammatory diseases, antioxidants have also demonstrated significant therapeutic potential in a variety of dermatological conditions. As previously reviewed, antioxidants have shown promise in managing skin disorders such as atopic dermatitis and psoriasis [91]. Beyond their role in alopecia areata, we also explore their relevance in androgenetic alopecia, vitiligo, and rheumatoid arthritis.

Recent research has increasingly recognized oxidative stress as a key contributor to various forms of hair loss, especially androgenetic alopecia (AGA). Although conventional agents such as minoxidil can promote hair growth, they often exhibit slow onset, limited efficacy, and insufficient ability to mitigate oxidative stress-induced follicular damage. In this context, Xiao et al. [17] proposed the use of nanomolybdenum, a transition metal-based antioxidant material, as a novel therapeutic approach. Due to its excellent electron transfer capabilities, nanomolybdenum effectively modulates local redox homeostasis, promotes follicular regeneration, and downregulates the expression of iNOS, COX-2, and androgen receptors, thereby accelerating the hair regeneration process. Moreover, its combination with minoxidil produced a synergistic effect, suggesting a potential therapeutic approach for nanomaterial-based antioxidant therapy in alopecia. Zhang et al. [92] further emphasized the role of oxidative stress in AGA by employing machine learning techniques to identify an efficient SOD-mimicking nanomaterial—manganese thiophosphate (MnPS_3_). This material exhibited a half-maximal inhibitory concentration (IC_50_) of just 3.61 μg/mL, outperforming most reported SOD-like nanozymes. They also developed MnPS_3_-loaded microneedle patches capable of penetrating the skin and targeting hair follicles, thereby effectively scavenging local ROS. Yang et al. [93] developed a multifunctional microneedle patch delivery system based on a curcumin–zinc metal–organic framework (ZnMOF-MN), which enables efficient transdermal delivery of Zn^2+^ and curcumin. This system exerts antioxidant, anti-apoptotic, and anti-androgenic effects, significantly enhancing dermal papilla cell activity and promoting hair follicle regeneration. Yang et al. [94] developed a quercetin–polydopamine-integrated nanoplatform (PDA@QLipo) designed to modulate the perfollicular microenvironment and stimulate follicle regeneration. This nanosystem not only exhibited potent ROS-scavenging activity but also promoted neovascularization, collectively contributing to a regenerative microenvironment favorable for hair growth. The pathogenesis of vitiligo involves oxidative stress as a major driving force behind melanocyte injury and programmed cell death. ROS (e.g., O_2_^−^ and H_2_O_2_) generated during melanin synthesis render melanocytes particularly vulnerable to oxidative injury [95]. A disrupted antioxidant system is evident in the affected skin of vitiligo patients, marked by elevated antioxidant enzyme activity (e.g., SOD) and impaired hydrogen peroxide detoxification. This redox imbalance may perpetuate melanocyte degeneration in depigmented lesions. To address this, Li et al. [96] developed a microneedle-delivered antioxidant and immunomodulatory nanodrug (PDA-JAKi) for targeted vitiligo therapy. This system consists of polydopamine nanoparticles loaded with the JAK inhibitor tofacitinib. The nanodrug alleviated ROS-induced melanocyte apoptosis and suppressed the release of self-antigens and subsequent immune activation. In vivo studies demonstrated its ability to restore pigmentation, reduce ROS accumulation, and improve tissue integrity, underscoring its dual antioxidant and immunomodulatory efficacy. In rheumatoid arthritis (RA), Han et al. [97] synthesized ultrasmall iron–quercetin coordination nanoclusters (Fe-Qur NCNs) to mitigate oxidative and inflammatory responses associated with RA. These nanoparticles retained the ROS-scavenging properties of quercetin while improving its water solubility and biocompatibility. In addition, excessive ROS were effectively neutralized by Fe-Qur NCNs, inhibited pro-inflammatory macrophage polarization, and suppressed NF-κB pathway activation, thereby reducing apoptosis and inflammatory cytokine release. In vivo, the nanodrug alleviated joint swelling and bone erosion, inhibited immune cell infiltration, promoted anti-inflammatory macrophage phenotypes, and attenuated osteoclast activity in RA mouse models. These results suggest that metal–natural product coordination nanoplatforms represent a promising strategy for managing RA and other oxidative stress-related diseases. In summary, oxidative stress contributes critically to the development of diverse cutaneous and immune-mediated inflammatory conditions. Nanomaterial-based antioxidant strategies, owing to their excellent targeting ability and biocompatibility, not only offer standalone therapeutic benefits but also demonstrate synergistic effects when combined with conventional treatments, highlighting their significant potential for clinical translation and further research.

## 7. Discussion

The treatment of IMIDs remains a significant challenge in both clinical and translational medicine, primarily due to their heterogeneous pathogenesis, chronic progression, and frequent systemic involvement [98]. IMIDs encompass a wide spectrum of disease types, including autoimmune disorders such as AA and MS, acute inflammatory injuries like AKI and ALI, and chronic inflammatory conditions such as IBD and periodontitis. A unifying pathological feature among these diseases is the excessive production of ROS, which not only directly damages tissues but also exacerbates immune dysregulation, thereby perpetuating a vicious cycle of inflammation [99,100]. Consequently, targeting oxidative stress has emerged as a theoretically grounded and promising therapeutic strategy.

However, the clinical efficacy of conventional antioxidant therapies remains limited, largely due to inherent shortcomings such as poor pharmacokinetics, inadequate tissue targeting, rapid metabolic degradation, and low intracellular retention. To address these limitations, recent research has increasingly focused on the development and application of nano-antioxidants. These nanomaterials or nano-delivery systems are engineered to exert direct or indirect antioxidative effects and demonstrate superior therapeutic performance. Nano-antioxidant platforms include metal oxide nanoparticles (e.g., zinc oxide, manganese dioxide), carbon-based nanomaterials (e.g., graphene oxide, fullerenes), polymeric nanoparticles, and lipid-based or exosome-mimetic delivery vehicles [101,102]. Compared to traditional antioxidants, these nanostructures offer several advantages, including enhanced stability in physiological environments, improved cellular uptake, targeted delivery to sites of inflammation or oxidative stress, and the ability to achieve sustained, localized drug release to maximize therapeutic efficacy. Furthermore, many nano-antioxidants possess intrinsic ROS-scavenging capabilities without the need for additional drug loading, thereby minimizing systemic exposure and reducing potential side effects [103]. Although nanoparticles or metallic nanoparticles (mNPs) are known to induce oxidative stress through excessive generation of ROS, recent advancements in nanoparticle engineering have demonstrated their potential to mitigate oxidative damage. Green-synthesized and surface-modified mNPs—such as plant-derived Cu and Pt nanoparticles—can scavenge free radicals via enzyme-mimetic activities resembling catalase or superoxide dismutase. These nano-antioxidants downregulate key pro-oxidant signaling pathways (e.g., NF-κB, JAK/STAT) and enhance cellular antioxidant defense mechanism [104]. Additionally, PEGylation and conjugation with natural antioxidants (e.g., flavonoids, polyphenols) offer effective strategies to reduce immune recognition and ROS-induced toxicity. Thus, depending on their composition and surface chemistry, mNPs can function either as pro-oxidant or antioxidant agents, providing a versatile platform for therapeutic modulation of oxidative stress [105].

## 8. Conclusions

An increasing body of evidence from in vitro studies, animal models, and early translational investigations suggests that nano-antioxidants can effectively alleviate inflammation, preserve tissue integrity, and modulate immune responses. For instance, in autoimmune diseases like AA, nano-antioxidants have been shown to mitigate inflammation by scavenging superoxide anions and hydrogen peroxide, offering a novel therapeutic avenue [17]. In MS models, polymeric nanoparticles loaded with antioxidants can traverse the blood–brain barrier and precisely deliver therapeutic agents to neuroinflammatory sites, thereby reducing demyelination and preserving neurological function [106]. In acute inflammatory conditions such as AKI, nano-antioxidants help alleviate renal oxidative stress, maintain mitochondrial function, and potentially enhance treatment outcomes [107]. Similarly, in chronic inflammatory diseases like IBD and ulcerative colitis, these agents promote mucosal healing, modulate gut microbiota composition, and reduce inflammatory cell infiltration in the colon [108].

Despite these encouraging preclinical findings, the clinical translation of nano-antioxidants remains in its infancy and faces several critical challenges. First, the biocompatibility and long-term safety of various nanomaterials are not yet fully understood, particularly under conditions of repeated or prolonged use. Second, the heterogeneity of IMIDs necessitates precision medicine approaches, as disease-specific redox pathways, immune activation patterns, and tissue microenvironments may significantly influence the efficacy and biodistribution of nano-antioxidants. Third, practical issues such as scalable manufacturing, regulatory approval, and cost-effectiveness must be systematically addressed. Moreover, nano-antioxidants must demonstrate therapeutic efficacy that is superior to—or at least non-inferior to—current standard treatments in rigorously designed randomized controlled clinical trials before widespread adoption can be recommended. Future research should prioritize the development of “smart” nano-antioxidant systems capable of responding to disease-specific microenvironmental cues (e.g., pH, ROS levels, enzymatic activity) to achieve precise and controlled drug release. Combining nano-antioxidant therapy with other modalities, such as immunomodulators, gene editing technologies (e.g., CRISPR/Cas9), or cell-based therapies, may further enhance therapeutic outcomes through synergistic effects. Additionally, personalized nanomedicine strategies—tailoring antioxidant interventions based on an individual’s disease phenotype, genetic markers, and microbiome characteristics—hold promise for improving treatment precision and minimizing adverse effects. In summary, nano-antioxidants represent a highly promising and multifunctional therapeutic platform for the management of diverse IMIDs. By overcoming the limitations of traditional antioxidants and leveraging the unique properties of nanomaterials, this strategy offers novel avenues for the targeted and sustained regulation of redox balance and immune function. Although substantial progress has been made, multidisciplinary collaboration and robust clinical validation are essential to fully realize their clinical potential. If successfully translated, nano-antioxidant therapies may substantially transform the therapeutic landscape of IMIDs and bring new hope to affected patients.

## Figures and Tables

**Table 1:** A brief summary of autoimmune disease treatment based on nano-antioxidant strategies.

Diseases	Active Ingredient	Nanomaterial	Side Effect	Benefit	Ref.
Alopecia areata (AA)	Minoxidil	Molybdenum nanoparticles (Mo NPs)	Mo-NPs may cause liver and kidney toxicity, oxidative imbalance, and potential inflammatory responses	Offering a novel direction and translational potential for antioxidant-based treatment of AA	[17]
Multiple Sclerosis (MS)	Curcumin	Dendritic nanocarriers (DNCs)	High doses (20 µM) reduce neural stem cell viability and increase oxidative stress, shifting differentiation toward astrocytes instead of oligodendrocytes	Enhancing remyelination capacity in vivo	[18]
	BBN-IONPs	Iron oxide nanoparticles	May not adequately reflect long-term effects, with the absence of human trials restricting therapeutic relevance	Elevated glutathione levels and total antioxidant capacity in brain tissues, reduced lipid peroxidation and inflammatory mediators	[19]
	MgH_2_	Low-toxicity magnesium hydride nanoparticles (MgH_2_)	Uncertainties remain regarding mechanism, brain region sensitivity, and variable microglial polarization effects	Significantly alleviating anxiety- and depression-like behaviors and attenuating inflammatory demyelinating lesions	[20]
	Clobetasol propionate (CP)	Double-layered nanoemulsion	Serious side effects include osteoporosis, glaucoma, and adrenal suppression	Improving brain-targeted delivery and reducing systemic toxicity	[21]
	Selenium and crocin	Crocin–selenium nanoparticles (Cor@SeNs)	Headache in two patients; no severe adverse events observed, generally well tolerated	Ameliorating MS-associated cognitive impairment	[22]
	Methylprednisolone, neutrophil membranes	Biomimetic nanosystem (TFMN)	Negligible cytotoxicity up to 200 μg/mL; no severe adverse effects observed in vivo, but long-term safety unknown	Indicating excellent safety and therapeutic potential	[23]

Abbreviation: BBN-IONPs, BBN loaded within iron oxide nanoparticles.

**Table 2:** A brief summary of acute inflammatory disease treatment based on nano-antioxidant strategies.

Diseases	Active Ingredient	Nanomaterial	Side Effect	Benefit	Ref.
Acute kidney injury	Cerium and luteolin	Cerium–luteolin coordination nanocomposite (CeLutNCs)	Excellent biocompatibility with no significant toxicity or adverse effects in vitro and in vivo	Significantly improved organ function and reduced oxidative stress and inflammation	[24]
	L-serine	L-serine (Rh-Ser, 2–4 nm)	Rh-Ser showed no obvious adverse reactions or systemic toxicity in the experiment	Mimicked multiple antioxidant enzymes to efficiently eliminate RONS	[25]
	Tetrahedral framework nucleic acid (tFNA)	Nanodrug delivery system (TTC)	tFNAs showed good biocompatibility with no nephrotoxicity or significant side effects in vitro and in vivo	Significantly ameliorated renal injury and restored renal function, indicating promising antioxidant-mediated therapeutic effects	[26]
	Epigallocatechin gallate (EGCG) and 5-aminosalicylic acid (5-ASA)	pH-stable antioxidant nanoparticle system (EGA NPs)	EGCG shows poor stability in acidic environments, limiting oral bioavailability	Effectively scavenged ROS and mitigated oxidative stress	[27]
	ethyl caffeate and 4-hydroxybenzyl alcohol	Supramolecular nanoplatform (Ser-HPEC)	No significant systemic side effects in vivo in the experiment	Preserved renal structure and function	[28]
Acute liver injury	Polyphenol	Phlorotannin-based nanoparticles (PT NPs)	Traditional polyphenols face bioavailability and immunogenicity issues	Effectively alleviated oxidative and inflammatory damage	[29]
	Catechin	CCN150	CCN150 showed negligible acute toxicity, but potential long-term risks like	Reduced systemic inflammation driven by ROS	[30]
	NO	BSA-SNO NPs	No significant toxicity observed; potential risks include immune response, NO over-release, or thiol oxidation/cross-linking	Alleviated oxidative stress, improved hepatic microcirculation	[31]
	phenylboronic-acid-protected L-DOPA precursor (PAD)	Responsive biomimetic antioxidant nanoplatform (PADN)	Potential risks include batch variability, incomplete H_2_O_2_ scavenging, and reactive group-related off-target effects	Integrated efficient ROS scavenging, inflammation suppression, and hepatocyte protection	[32]

Abbreviation: CCN150, catechin-condensed nanotherapeutics; NO, nitric oxide; BSA, serum albumin; BSA-SNO NPs, self-sulfhydrated, nitro-fixed albumin nanoparticles.

**Table 3:** A brief summary of chronic inflammatory disease healing based on nano-antioxidant strategies.

Diseases	Active Ingredient	Nanomaterial	Side Effect	Benefit	Ref.
Periodontitis	quercetin	A mesoporous bioactive glass-based nanocarrier system	No significant toxicity observed; potential risks include initial burst release and uncertain long-term local effects.	Promoted both osteogenesis and angiogenesis in experimental periodontitis-related bone defects	[33]
	melatonin (MT)	melatonin-derived carbon dots (MT-CDs)	No significant side effects were observed during the experiment	Reduced alveolar bone resorption and osteoclast activity	[34]
	melanin	P/D-MNP-Ag	No significant organ inflammation observed in the experiment	Disrupted biofilms, attenuated inflammation, and reduced alveolar bone loss	[35]
	HAS, Mn^2+^, [Fe(CN)6]^4−^	HSA-MDSPB NPs	No significant side effects were observed during the experiment	Broadly scavenged ROS species	[36]
Ulcerative colitis (UC)	somatostatin (SST) and manno-oligosaccharide (MOS)	EUP-SeNPs	No obvious toxicity observed, potential risks include selenium accumulation	Improved oral stability and intestinal localization	[37]
	gallic acid	thiolated anionic nanoliposomes	No apparent toxicity observed	Prolonged drug residence time and enhanced local therapeutic effects	[38]
	curcumin	CMC	No obvious toxicity or systemic side effects were observed in this experiment	Effectively scavenged ROS, restored mucosal integrity, and regulated gut microbiota composition to mitigate UC pathology	[39]
	PSB	PSB@NP-FA	Minimized systemic toxicity compared to free DEX	Modulated dendritic cell function, promoted M2 polarization of macrophages, and regulated T cell infiltration	[40]
	TMCs and PEG	PMNFs	No significant side effects	Increased junction protein and MUC2 expression, thereby preserving intestinal barrier function	[41]
	pterostilbene	HA-L-Arg-CO_2_@NPs	No significant side effects	Downregulated inflammatory mediators, restored barrier permeability, and effectively alleviated UC symptoms	[42]
	carbon dots	MML-CDs	No significant side effects were observed during the experiment	Demonstrated promising therapeutic efficacy	[43]

Abbreviation: P, phosphate group; D, 2-(Dimethylamino)ethyl methacrylate; MNP-Ag, melanin-Ag; HSA-MDSPB, human serum albumin-crosslinked, manganese-doped, self-assembling Prussian blue; CMC, carboxymethyl cellulose; PSB, pterostilbene; NP, nanoparticle; FA, folic acid; TMCs, transition metal chalcogenides; PEG, polyethylene glycol; PMNFs, PEG-modified Mo3Se4 nano flakes; HA-L-Arg-CO_2_@NPs, hyaluronic acid (HA)-modified L-arginine CO_2_ nanoparticles; MML-CDs, carbon dots derived from magnetite and medicated leaven.

## Data Availability

No new data were created in this study. Permission for re-use of previously published figures has been obtained.
